# Factors Associated with Attitudes towards Seasonal Influenza Vaccination in Poland: A Nationwide Cross-Sectional Survey in 2020

**DOI:** 10.3390/vaccines9111336

**Published:** 2021-11-17

**Authors:** Piotr Samel-Kowalik, Mateusz Jankowski, Mira Lisiecka-Biełanowicz, Aurelia Ostrowska, Mariusz Gujski, Bartosz Kobuszewski, Jarosław Pinkas, Filip Raciborski

**Affiliations:** 1 Department of Prevention of Environmental Hazards and Allergology, Medical University of Warsaw, 02-091 Warsaw, Poland; piotr.samel@wum.edu.pl (P.S.-K.); mira.bielanowicz@wum.edu.pl (M.L.-B.); filip.raciborski@wum.edu.pl (F.R.); 2 School of Public Health, Centre of Postgraduate Medical Education, 01-826 Warsaw, Poland; aostrowska@cmkp.edu.pl (A.O.); bkobuszewski@cmkp.edu.pl (B.K.); jpinkas@cmkp.edu.pl (J.P.); 3 Department of Public Health, Medical University of Warsaw, 02-097 Warsaw, Poland; mariusz.gujski@wum.edu.pl

**Keywords:** influenza, influenza vaccine, vaccine hesitancy, vaccines, Poland

## Abstract

We aimed to assess attitudes towards the influenza vaccine and factors associated with a willingness to vaccinate against seasonal influenza in Poland during the COVID-19 pandemic (flu season 2020/2021). This cross-sectional questionnaire-based study was carried out between 5 and 15 November 2020 on a representative nationwide sample of 1052 individuals aged 18+ in Poland. Of the respondents, 5.5% (95% CI: 4.3–7.0%) declared that they had already got vaccinated against influenza and 13.4% (95% CI: 11.4–15.6%) declared a willingness to vaccinate against influenza during the 2020/2021 season. Out of nine different factors analyzed in this study, only three were significantly associated with attitudes towards influenza vaccination. Participants aged 75 years and over (OR = 5.82; 95% CI: 2.63–12.85), as well as participants aged 60–74 years (OR = 2.43; 95% CI: 1.30–4.54), compared to those aged 19–29, had significantly higher odds of having a positive attitude towards seasonal influenza vaccination. Respondents who define themselves as completely religious unbelievers (OR = 4.34; 95% CI: 1.79–10.55), as well as Internet users (OR = 2.12; 95% CI: 1.30–3.47), had higher odds of having a positive attitude towards influenza vaccination. Despite the COVID-19 pandemic, the percentage of adults in Poland who already got vaccinated or declared a willingness to vaccinate against influenza remains low. This also applies to high-risk groups.

## 1. Introduction

Seasonal influenza is an acute respiratory infection caused by influenza viruses (types A, B, C, and D) [1]. Influenza spreads easily from person to person, mostly by droplets and aerosols from infected people [2]. Transmission is especially high in crowded areas including schools and nursing homes [3]. The incubation period is one to four days [2]. Symptoms are usually mild and limited to the upper respiratory tract [1,2]. However, some groups are at higher risk of severe illness, including young children, pregnant women, older adults, and those with chronic medical conditions [4]. According to the World Health Organization (WHO), annually, influenza viruses infect between 5% and 15% of the global population, causing 3–5 million cases of severe illness and up to 500,000 deaths [1,5].

Vaccination is the most effective measure to prevent influenza [1,6]. Vaccination against seasonal influenza reduces sickness, medical visits, hospital admissions, and deaths [6]. Because influenza viruses change rapidly due to antigenic drift, influenza vaccines are reformulated and delivered annually [7]. Vaccines include inactivated or live-attenuated influenza type A and B viruses, with three or four subtypes per vaccine [7]. The WHO updates recommendations for the composition of the influenza vaccine twice a year, but countries make decisions on the administration of influenza vaccines based on local disease burden, available resources, and capacity [1,7]. 

WHO recommends annual vaccination for pregnant women, children aged between 6 months and 5 years, adults aged more than 65 years, individuals with chronic medical conditions, and healthcare workers [1]. The European Centre for Disease Prevention and Control (ECDC) recommends vaccination for the elderly as a priority, with a secondary priority for people with chronic medical conditions and healthcare workers [8]. However, seasonal influenza vaccination recommendations vary by country [9]. Findings from the study on seasonal influenza immunization policies (2017–2018) in the European Union (EU) and European Economic Area (EEA) member states showed differences in the vaccination recommendation [9]. Out of 30 member states, 6 recommended vaccinations for children/adolescents <18 years of age and 28 recommended vaccination of pregnant women [9]. All member states recommended seasonal influenza vaccination for older age groups, although with different age thresholds. 

The ECDC encourages member states to adopt and implement action plans aimed at reaching seasonal influenza vaccination coverage among older age groups of 75% [8]. However, retrospective analysis of vaccination coverage rates in 19 member states in 2016–2017 showed that the vaccination coverage rates in older target populations ranged from 2.0% to 72.8% (median 47.1%). The highest vaccination coverage rate was observed in the United Kingdom and the lowest in Estonia, Latvia, and Poland [9]. 

Epidemiological data indicate low and declining use of seasonal influenza vaccines in the WHO European Region [10]. Reduced influenza vaccination uptake may be attributed to decreased vaccine confidence level, lack of recommendation from healthcare providers, financial barriers (e.g., out-of-pocket costs to receive vaccination) as well as system barriers (e.g., lack of access to vaccine or the need to independently purchase and deliver the vaccine to the healthcare facility) [11]. 

Poland is a country with one of the lowest influenza vaccination coverage rates among the EU member states [9]. The study conducted on a representative sample of adult Polish residents showed that in November 2016, 6% of respondents declared that they had received an influenza vaccine and another 7% declared a willingness to vaccinate against influenza [12]. Three years earlier (in 2013), similar results were obtained, 7% and 6%, respectively [13]. Between influenza season 2001/2002 and 2016/2017, the vaccination coverage rate in the general population decreased from 10.57% to 3.67% [14]. In Poland, influenza vaccination is voluntary. Public health authorities recommend vaccination for pregnant women, children aged between 6 months and 18 years (with particular emphasis on children aged between 6 months and 5 years), adults aged 55 and over, individuals with chronic medical conditions, residents of long-term care facilities, selected professional groups (uniformed services, trade workers, transport sector workers, teachers, and social workers), healthcare workers, and medical students [15]. 

In Poland, influenza vaccines are dispensed mainly in pharmacies based on a medical prescription. The vaccine prescription is most often issued by the family doctor, who usually carries out the vaccination. Therefore, family doctors play a key role in advocating for influenza vaccination [16]. People who are covered by the free vaccination program (including medical staff, pharmacists, teachers, seniors, and residents of nursing homes) can go directly to a dedicated vaccination point and do not require a prescription. Before 2018, the influenza vaccine was self-paid (out-of-pocket) in all groups. To reduce financial barriers, in July 2018, public health authorities introduced the influenza vaccine reimbursement for selected high-risk groups (50% reimbursement for adults aged 65 and over) [17]. In 2020, the increase in seasonal influenza vaccination coverage among older age groups, because of the elimination of the financial barrier, resulted in the extension of the scope of the reimbursement to pregnant women (50% reimbursement), people over 75 (100% reimbursement), children between 24 months and 5 years, and adults aged 18–65 years with chronic medical conditions [17]. As of 2021, influenza vaccination is free of charge for pregnant women [17]. 

In addition to the financial, systems, and educational barriers, the seasonal influenza vaccination coverage rate depends on individual attitudes towards the influenza vaccine. Most of the studies on attitudes towards influenza vaccination in Poland were carried out among patients with chronic medical conditions or healthcare professionals/medical students [18,19,20,21]. There is a limited amount of up-to-date data on the attitudes towards influenza vaccination in a representative sample of adults in Poland. Moreover, the impact of the COVID-19 pandemic on attitudes towards influenza vaccination has not yet been fully understood. Identification of the factors associated with attitudes towards the seasonal influenza vaccine will allow for the implementation of educational activities that build confidence in vaccination and increase influenza vaccine uptake in Poland.

We aimed to assess the attitudes towards the influenza vaccine and factors associated with a willingness to vaccinate against seasonal influenza in Poland during the COVID-19 pandemic (flu season 2020/2021).

## 2. Materials and Methods

### 2.1. Study Design and Population

This cross-sectional study was carried out between 5 and 15 November 2020 on a representative nationwide sample of 1052 individuals aged 18+ in Poland. Data purchased for this secondary statistical analysis were driven from a cross-sectional questionnaire survey carried out by a specialized company—Public Opinion Research Center (CBOS). The Public Opinion Research Center (CBOS) is one of the most popular companies in Poland that conducts national cross-sectional surveys on the population’s opinion regarding different social issues [22]. 

The sampling frame is based on demographic data from the Universal Electronic System for Registration of the Population (PESEL) registry on the number of residents in all localities of the country, broken down by age and sex [22,23] (CBOS is allowed to use the PESEL resources for public opinion polls due to the provisions of two acts: the Act on Population Records and the Act on Personal Data Protection). Computer-assisted personal interviewing (CAPI; 43% of respondents), computer-assisted telephone interviewing (CATI; 42.4% of respondents), and computer-assisted web interviewing (CAWI; 14.6% of respondents) were used.

According to the provisions of the law in force in Poland, CBOS applies to the administrator of the PESEL database with a request to draw a representative sample of adult residents of the country to conduct a survey. Respondents are selected from all strata. CBOS introduces the division into layers to gain more control over the territorial dispersion of the sample. The studied population is divided according to the categories of the place of residence. The allocation is prepared to obtain a sample in which each layer is represented proportionally to its size. The final stage of sampling is drawing. It takes place in two stages. The first involves drawing of the localities in each layer. The number of randomly selected localities is proportional to the size of the stratum, localities are drawn with a probability proportional to the number of inhabitants. If the layer consists of one locality, the first stage is skipped. In the next step, exactly 10 people are drawn from each town (or a multiple of this number if the town was selected several times) belonging to the studied population of adult Polish citizens. In this way, we get personal samples, i.e., samples consisting of precisely defined people.

CBOS send an announcement letter to the selected people, in which, among other things, they explain and inform that the selected person is not obligated to participate in the study, what the legal grounds are for conducting this type of research, and the respondent’s rights related to the General Data Protection Regulation (GDPR; we meet the so-called information obligation according to Articles 13 and 14 of the GDPR). Consent to participate in the survey is given by the potential respondent when he or she agrees to accept the interviewer, give a telephone interview, or complete an online survey by himself.

After each survey, weighting of the results is prepared according to the data of the Central Statistical Office and demographic data from the PESEL register. For weighting, the following characteristics of the respondents are included: gender, city/village class, voivodship of residence, age group, education level, and social and professional group.

### 2.2. Measures

The study was conducted in November 2020. Questions on the COVID-19 pandemic, attitudes towards the COVID-19 vaccine, attitudes towards seasonal influenza vaccine, and the willingness to get vaccinated were used in the questionnaire. Questions also addressed personal characteristics, including age, sex, size of place of residence, educational level, occupational status, financial status, religious faith, and Internet use. Details are presented in Supplementary Material Table S1 (Translated version of the study questionnaire).

Uptake of the influenza vaccine was defined using the question: “Have you been vaccinated against influenza this fall/winter season? (Yes/No/Refusal to answer)”. Willingness to vaccinate against seasonal influenza was defined using the question: “Are you going to have influenza vaccine this fall/winter season? (Yes/No/Refusal to answer)”. Respondents who got vaccinated or declared a willingness to vaccinate against influenza during the 2020/2021 were classified as those who declare positive attitudes towards seasonal influenza vaccination. Attitudes towards using the Internet were defined using the question: “Do you have an e-mail that you check regularly—i.e., at least 3–4 times a week? (Yes/No)”. The analyses presented in this article are based on affirmative responses (“yes”)—either being vaccinated or willing to be vaccinated. With this approach, refusals to answer were also treated with a negative answer.

### 2.3. Statistical Analysis 

The data were analyzed with SPSS version 27 (Armonk, NY, USA: IBM Corp) and R version 4.0.4 (R Foundation for Statistical Computing, Vienna, Austria, 2021). Demographic weighting was applied. The distribution of categorical variables was shown by proportions. Statistical testing to compare categorical variables was completed using the independent samples chi-square test. Associations between sociodemographic factors (gender, age, place of residence, educational level, occupational status, self-reported financial status, having children under 18 years of age, participation in religious practices, and using the Internet) and positive attitudes towards seasonal influenza vaccination were analyzed using the multivariate logistic regression analyses. The strength of association was measured by the odds ratio (OR) and 95% confidence intervals (CI). The level of statistical significance was set at alfa 0.05

### 2.4. Ethics 

This study was performed in line with the principles expressed in the Declaration of Helsinki. Data purchased for the purposes of this secondary statistical analysis were collected by a specialized survey company—Public Opinion Research Center (CBOS). Datasets were anonymous and prevented the identification of any individual study subject by the research team at any stage of the study. 

## 3. Results

### 3.1. Characteristics of the Study Population

The analysis was based on responses of 1052 individuals (52.9% females). The mean age was 48.9 (±17.8) years. Table 1 shows the demographic characteristics of the study sample.

### 3.2. Percentage of Respondents Who Got Vaccinated or Declared a Willingness to Vaccinate against Influenza during the 2020/2021 Season by Socio-Demographic Factors 

**Figure 1:**
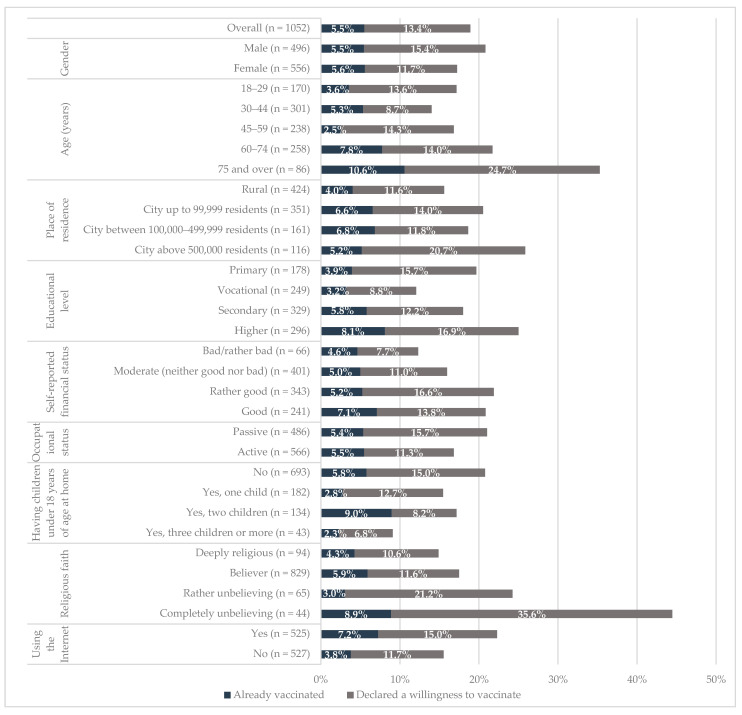
Percentage of respondents who got vaccinated or declared a willingness to vaccinate against influenza during the 2020/2021 season by socio-demographic factors, Poland, November 2020.

In November 2020, of the 1052 respondents, 5.5% (95% CI: 4.3–7.0%) declared that they already got vaccinated against influenza during the 2020/2021 season. Moreover, 13.4% (95% CI: 11.4–15.6%) declared a willingness to vaccinate against influenza during the 2020/2021 season (Figure 1).

In bivariate analyses (Figure 1), there were significant differences (*p* < 0.05) in the percentage of respondents who got vaccinated against influenza during the 2020/2021 season by age and using the Internet. There were no significant differences in the percentage of respondents who got vaccinated against influenza by gender (*p* = 0.933), place of residence (*p* = 0.353), educational level (*p* = 0.061), self-reported financial status (*p* = 0.699), occupational status (*p* = 0.918), number of children at home (*p* = 0.088), and religious faith (*p* = 0.522).

There were significant differences (*p* < 0.05) in the percentage of respondents who declared a willingness to vaccinate against influenza during the 2020/2021 season by age (*p* < 0.01), educational level (*p* < 0.05), occupational status (*p* < 0.05), and religious faith (*p* < 0.001). There were no significant differences in the percentage of respondents who declared a willingness to vaccinate against influenza by gender (*p* = 0.9082), place of residence (*p* = 0.072), self-reported financial status (*p* = 0.072), number of children at home (*p* = 0.094), and attitudes towards using the Internet (*p* = 0.118). 

### 3.3. Percentage of Respondents Who Declared Positive Attitudes towards Seasonal Influenza Vaccination by Socio-Demographic Factors 

In total, 18.9% (95% CI: 16.5–21.3%) of respondents declared positive attitudes towards seasonal influenza vaccination (respondents who got vaccinated or declared a willingness to vaccinate against influenza). The percentage of respondents who declared positive attitudes towards seasonal influenza vaccination increased with age (*p* < 0.001). The percentage of respondents who declared positive attitudes towards seasonal influenza vaccination was more than two times higher among those with higher education compared to respondents with vocational education (25.0 vs. 12.1%; *p* < 0.01). The highest percentage of respondents who declared positive attitudes towards seasonal influenza vaccination was almost three times higher among those who define themselves as complete religious unbelievers, compared to deeply religious respondents (44.4 vs. 15.1%; *p* < 0.001). Internet users more often declared positive attitudes towards seasonal influenza vaccination compared to those respondents who do not use the Internet (22.2 vs. 15.6%; *p* < 0.01). Details are presented in Table 2.

### 3.4. Perception of the COVID-19 Pandemic and Attitudes towards Seasonal Influenza Vaccination

There were significant differences in the attitudes towards seasonal influenza vaccination depending on the perception of the COVID-19 pandemic (Table 3). The percentage of respondents who declared positive attitudes towards seasonal influenza vaccination was almost four times higher among those who showed high levels of fear of COVID-19 compared to respondents who declared no fear of coronavirus infection (27.0 vs. 7.6%; *p* < 0.001). The lowest percentage of respondents who declared positive attitudes towards seasonal influenza vaccination was among those who respondents who believed in conspiracy theories and fake news about the COVID-19 pandemic. Among those who believed that pharmaceutical lobbies, politicians, and the media around the world are deliberately exaggerating the dangers of the coronavirus, only 3.5% declared positive attitudes towards seasonal influenza vaccination. Respondents who declared lack of willingness to vaccinate against COVID-19 were also not willing to vaccinate against seasonal influenza. Details are presented in Table 3.

### 3.5. Factors Associated with Positive Attitudes towards Seasonal Influenza Vaccination

**Figure 2:**
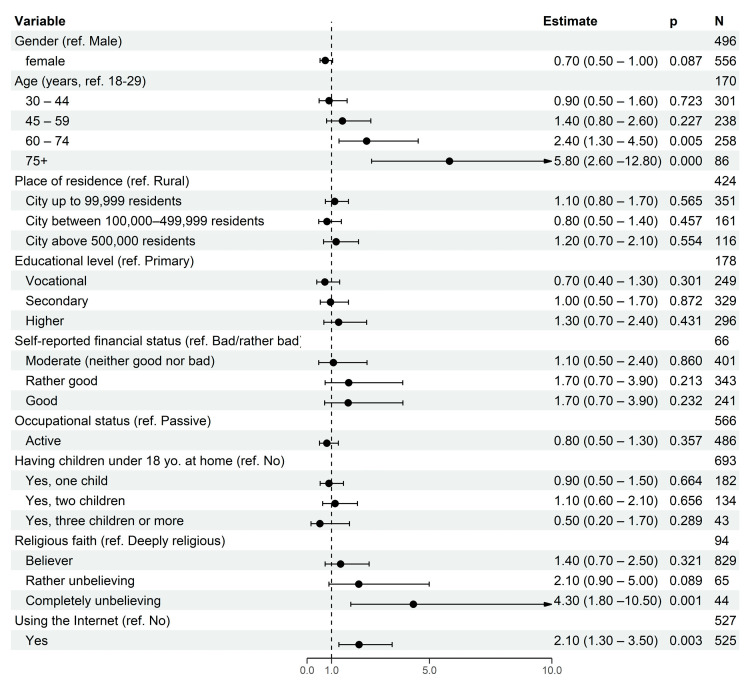
Socio-demographic factors associated with positive attitudes towards seasonal influenza vaccination—the multivariate logistic regression model.

The multivariate logistic regression which explained the positive attitudes towards seasonal influenza vaccination obtained a Cox and Snell R-Squared value of 0.079 and Nagelkerke R-Squared of 0.127. All analyzed variables were included in the model (Figure 2). Participants aged 75 years and over compared to those aged 19–29 years had almost six times higher odds of having a positive attitude towards seasonal influenza vaccination (OR = 5.82; 95% CI: 2.63–12.85). Moreover, participants aged 60–74 years had more than two times higher odds of having a positive attitude towards seasonal influenza vaccination (OR = 2.43; 95% CI: 1.30–4.54) compared to those aged 19–29 years. Respondents who define themselves as complete religious unbelievers compared to deeply religious respondents had more than four times higher odds of having a positive attitude towards seasonal influenza vaccination (OR = 4.34; 95% CI: 1.79–10.55). Internet users had more than two times higher odds of having a positive attitude towards seasonal influenza vaccination (OR = 2.12; 95% CI: 1.30–3.47) compared to those who do not use the Internet. Details are presented in Figure 2. 

## 4. Discussion

To the best of the authors’ knowledge, this is one of the most up-to-date studies on attitudes towards influenza vaccines in Poland carried out during the COVID-19 pandemic (flu season 2020/2021). In 2020, the public debate focused on the COVID-19 pandemic [24]. Tips on respiratory infection prevention methods were widely reported in the media. As of November 2020, when this study was conducted, the COVID-19 vaccine was not yet available. Vaccination against influenza may reduce burden of care for patients with respiratory tract infections. Growing awareness of infectious diseases can influence attitudes towards influenza vaccination. This study showed that in November 2020, among adults in Poland, 5.5% had already been vaccinated against influenza and 13.4% declared a willingness to vaccinate against influenza during the 2020/2021 season, which suggests an increased interest in influenza vaccination compared to previous years [14]. Out of nine different variables analyzed in the multivariate logistic regression model, only age, religious faith, and the use of the Internet were significantly associated with attitudes towards seasonal influenza vaccination.

The nationwide representative studies conducted in Poland before the COVID-19 pandemic in 2013 and 2017 show that 6–7% of people declare that they had been vaccinated against influenza in November of a given year and the same number declared such an intention [12,13]. Our analyses carried out on the material obtained with the same research method in November 2020, i.e., during the COVID-19 pandemic, showed similar results in terms of respondents who declared that they had received an influenza vaccine (5.5% in 2020). However, the proportion of people who declared a willingness to vaccinate against influenza in 2020 (13.4%) was approximately twice as high as in previous studies [12,13].

In the WHO European Region, annual influenza epidemics usually occur during fall and winter [10]. In Poland, in every influenza season, the first seasonal influenza vaccines are available in September. To reduce financial barriers, influenza vaccine refunds were introduced in 2018, including full refund in selected groups from 2020 [17]. To ensure access to vaccines, in recent years, public health authorities have increased the number of influenza vaccines ordered from manufacturers. In the last quarter of 2021, legislative work is also underway to enable pharmacists to perform influenza vaccinations at a pharmacy [25]. This change would remove one of the major systemic barriers and a person wishing to get vaccinated will not have to transport the vaccine to the doctor’s office by himself, which is time-consuming and poses the risk of breaking the “cold chain” [25]. 

It is estimated that during the 2020/2021 influenza season, approximately 6% of inhabitants of Poland got vaccinated against seasonal influenza [26]. In this study, in November 2020, 5.5% of adults in Poland declared that they already got vaccinated against influenza. However, 13.4% of adults declared a willingness to vaccinate against influenza during the 2020/2021 season. A comparison of self-reported declarations with market data indicates that a significant percentage of Poles willing to be vaccinated in the 2020/2021 influenza season did not do so [26]. We can hypothesize that such a large difference between the percentage of respondents who declared a willingness to vaccinate against influenza and the real number of those who got vaccinated may result from still existing financial, systemic, and educational barriers. In addition to providing access to vaccines, the possibility of vaccination close to home, and the removal of financial barriers, health policy programs should focus on education about the “patient path” and the removal of systemic barriers. 

In addition to activities at the national level carried out by public authorities (removing financial and systemic barriers), at the local level, family doctors play a key role in advocating for influenza vaccination [16,18]. Identification of the factors associated with attitudes towards the seasonal influenza vaccine will allow for the better implementation of educational activities and prepare personalized communication. 

This study showed that age was associated with higher odds of having a positive attitude towards seasonal influenza vaccination. Odds of having a positive attitude towards seasonal influenza vaccination significantly increased after 60 years of age. This observation is in line with global trends in seasonal influenza vaccination uptake [7,9,10]. In the EU/EEA region, the vaccination coverage rate among older adults is several times higher compared to the youngest groups [9]. This observation may result from the fact that older adults are a priority group listed in seasonal influenza vaccination recommendations [8,15]. Moreover, reimbursement of influenza vaccine may encourage older adults to vaccinate against influenza. 

In this study, a significant association between religious faith and attitudes towards influenza vaccination was observed. Respondents who define themselves as complete religious unbelievers had more than four times higher odds of having a positive attitude towards seasonal influenza vaccination compared to deeply religious respondents. The opposite association was reported in the study on attitudes towards the COVID-19 vaccine [27]. In April 2021, in a representative sample of adults in Poland, passivity towards participating in religious practices was significantly associated with a lack of willingness to vaccinate against COVID-19 [27]. In Poland, 92.9% of the population identified themselves with the Roman Catholic church [28]. We can hypothesize that the positive stance of religious authorities on vaccination against influenza may encourage believers to vaccinate [29]. This observation requires further investigation. 

The COVID-19 pandemic showed that vaccine disinformation spread through the Internet and social media [30]. Internet users may be exposed to content that undermines vaccine confidence [31]. A previous study reported that a lack of willingness to vaccinate against COVID-19 was associated with the use of the Internet [27]. However, the number of medical fake-news items about the influenza is much smaller than that of those about COVID-19. The Internet has become a major source of information for adolescent and adults. This finding indicates that public health authorities should include the Internet and social media as communication channels when designing educational campaigns [32].

This study was carried out during the second wave of the COVID-19 pandemic in Poland. The impact of the COVID-19 pandemic on attitudes towards infectious diseases and vaccinations is unknown. However, this study showed that the percentage of respondents who declared positive attitudes towards seasonal influenza vaccination was significantly higher among those who showed high levels of fear of COVID-19 and those who did not believe in conspiracy theories and fake news about the COVID-19 pandemic. Moreover, respondents who declared a lack of willingness to vaccinate against COVID-19 were also not willing to vaccinate against seasonal influenza. The impact of the COVID-19 pandemic on public attitudes towards vaccinations, especially childhood vaccination, requires further investigation. 

Findings from the studies on attitudes towards influenza vaccination among the patients, students, and healthcare workers in Poland showed that educational level, place of residence, and having children significantly may significantly affect the attitude toward influenza immunization [18,19,20]. In this study, there were no significant associations between gender, place of residence, educational level, occupational status, self-reported financial status, having children under 18 years of age, and attitudes towards influenza vaccination. In contrast to the studies cited above, this study was conducted in the general population (a representative sample of adult inhabitants of Poland). 

This study was conducted in November 2020. The first influenza vaccines are available from September. In Poland, due to geography, vaccination against influenza is recommended before the peak of the influenza season [33]. Vaccination should be carried out between September and December. 

This study has practical implications for healthcare professionals and policymakers. First, the percentage of respondents who declared positive attitudes towards influenza vaccination (got vaccinated or declared a willingness to vaccinate against influenza) during the 2020/2021 season was three times higher than the real number of those who got vaccinated based on the real-world/market data (18.9% vs. 6.03% [26]). Further activities are needed to ensure easy access to influenza vaccines for those individuals who intend to get vaccinated against influenza. Secondly, factors associated with attitudes towards seasonal influenza vaccination described in this study suggest that online educational campaigns should be launched. Moreover, encouraging religious organizations to support vaccination programs can increase the vaccination coverage rate.

This study has several limitations. First, we used secondary data on attitudes towards the influenza vaccine, so the scope of the analysis is limited to available data. When secondary data analysis is used, the researchers have no influence on the shape of the questionnaire and the process of collecting and controlling research material [34]. Therefore, it is crucial to verify the source of the data and the credibility of the institution from which they come. In some cases, limited or no access to audit documentation is also an issue. Secondly, individual motivations for avoiding influenza vaccination have not been assessed. Thirdly, this study was based on the results of a survey conducted in November 2020. Nevertheless, this is one of the most up-to-date studies on attitudes towards influenza vaccines in Poland carried out during the COVID-19 pandemic.

## 5. Conclusions

In November 2020, less than one-fifth of adult inhabitants of Poland got vaccinated against influenza or declared a willingness to vaccinate against influenza during the 2020/2021 season. Older age (60 years and over), passivity towards religious practices as well as being an Internet user were significantly associated with positive attitudes towards seasonal influenza vaccination. Moreover, respondents who declared negative attitudes towards the COVID-19 pandemic more often declared lack of willingness to vaccinate against seasonal influenza.

## Figures and Tables

**Table 1:** Characteristics of the study group (*n* = 1052).

Variable	*n*	%
Overall	1052	100
Gender		
Female	556	52.9
Male	496	47.1
Age (years)		
18–29	170	16.1
30–44	301	28.6
45–59	238	22.6
60–74	258	24.5
75 and over	86	8.2
Place of residence		
Rural	424	40.3
City up to 99,999 residents	351	33.4
City between 100,000–499,999 residents	161	15.3
City above 500,000 residents	116	11.0
Educational level		
Primary	178	16.9
Vocational	249	23.7
Secondary	329	31.3
Higher	296	28.1
Occupational status		
Active	566	53.8
Passive	486	46.2
Self-reported financial status		
Bad/rather bad	66	6.3
Moderate (neither good nor bad)	401	38.2
Rather good	343	32.6
Good	241	22.9
Having children under 18 years of age		
No	693	65.9
Yes, one child	182	17.3
Yes, two children	134	12.7
Yes, three children or more	43	4.1
Religious faith (*n* = 1032)		
Deeply religious	94	9.1
Believer	829	80.3
Rather unbelieving	65	6.3
Completely unbelieving	44	4.3
Using the Internet		
Yes	525	49.9
No	527	50.1

**Table 2:** Percentage of respondents who declared positive attitudes towards seasonal influenza vaccination by socio-demographic factors, Poland, November 2020.

Variable	*n*	% (95% CI)	*p*-Value
Overall	1052	18.9 (16.5–21.3)	
Gender			
Female	556	17.1 (14.1–20.4)	0.124
Male	496	20.8 (17.4–24.5)	
Age (years)			
18–29	170	17.4 (12.0–23.2)	<0.001
30–44	301	14.2 (10.7–18.6)	
45–59	238	16.5 (12.1–21.5)	
60–74	258	21.9 (17.4–27.4)	
75 and over	86	35.3 (25.4–45.3)	
Place of residence			
Rural	424	15.6 (12.4–19.2)	0.061
City up to 99,999 residents	351	20.6 (16.5–25.0)	
City between 100,000–499,999 residents	161	18.6 (13.2–25.2)	
City above 500,000 residents	116	25.8 (18.6–34.4)	
Educational level			
Primary	178	19.6 (14.3–26.0)	<0.01
Vocational	249	12.1 (8.4–16.5)	
Secondary	329	18.1 (14.1–22.3)	
Higher	296	25.0 (20.3–30.2)	
Occupational status			
Active	566	16.9 (14.0–20.2)	0.078
Passive	486	21.2 (17.7–25.0)	
Self-reported financial status			
Bad/rather bad	66	12.7 (5.9–21.6)	0.077
Moderate (neither good nor bad)	401	16.0 (12.6–19.8)	
Rather good	343	21.8 (17.7–26.5)	
Good	241	21.0 (16.4–26.6)	
Having children under 18 years of age at home			
No	693	20.7 (17.9–23.9)	0.064
Yes, one child	182	15.6 (10.7–21.2)	
Yes, two children	134	17.2 (11.5–24.2)	
Yes, three children or more	43	7.8 (2.0–17.5)	
Religious faith			
Deeply religious	94	15.1 (8.8–23.1)	<0.001
Believer	829	17.5 (15.0–20.2)	
Rather unbelieving	65	24.0 (15.4–36.0)	
Completely unbelieving	44	44.4 (31.4–60.1)	
Using the Internet			
Yes	525	22.2 (18.7–25.8)	<0.01
No	527	15.6 (12.7–18.8)	

**Table 3:** Percentage of respondents who declared positive attitudes towards seasonal influenza vaccination by self-reported attitudes towards the COVID-19 pandemic, Poland, November 2020.

Variable	*n*	% (95% CI)	*p*-Value
Overall	1052	18.9 (16.5–21.3)	
Are you personally afraid of coronavirus infection?			
Yes, I am very afraid	304	27.0 (22.2–32.2)	<0.001
Yes, I’m a little scared	424	19.4 (15.8–23.3)	
No, I’m not afraid	189	12.2 (8.1–17.4)	
No, I’m not afraid at all	121	7.6 (3.7–13.1)	
Hard to say	12	14.6 (3.6–43.6)	
In your opinion, do you think the coronavirus epidemic for the health of Poles is?			
A real threat	633	25.0 (21.7–28.4)	<0.001
An exaggerated threat	331	8.9 (6.1–12.2)	
A fictional threat at all	34	1.8 (0.3–12.9)	
Hard to say	54	18.8 (9.9–30.4)	
Pharmaceutical lobbies, politicians, and the media around the world are deliberately exaggerating the dangers of the coronavirus.			
I definitely agree	164	3.5 (1.5–7.4)	<0.001
I tend to agree	311	13.2 (9.8–17.3)	
I rather disagree	239	22.2 (17.3–27.8)	
I strongly disagree	220	34.9 (28.9–41.5)	
Hard to say	117	17.4 (11.1–24.7)	
The coronavirus pandemic was artificially triggered to reduce the population of humanity on Earth.			
I definitely agree	85	10.9 (5.4–18.4)	<0.001
I tend to agree	213	10.2 (6.8–14.9)	
I rather disagree	273	16.1 (12.1–20.8)	
I strongly disagree	287	33.3 (27.8–38.7)	
Hard to say	193	14.2 (9.6–19.4)	
If a COVID-19 vaccine was available, would you get vaccinated against COVID-19?			
I definitely agree	168	53.3 (45.4–60.4)	<0.01
I tend to agree	208	29.6 (23.5–35.8)	
I rather disagree	208	6.2 (3.5–10.2)	
I strongly disagree	287	3.5 (1.8–6.1)	
Hard to say	179	13.4 (9.0–19.0)	

## Data Availability

The datasets generated during and/or analyzed during the current study are available from the corresponding author on reasonable request.

## Supplementary Materials

The following are available online at https://www.mdpi.com/article/10.3390/vaccines9111336/s1, Table S1: Translated version of the study questionnaire.
