# Supplementary material for: Factors Associated with Attitudes towards Seasonal Influenza Vaccination in Poland: A Nationwide Cross-Sectional Survey in 2020
Source: Vaccines (Basel). 2021 Nov 17;9(11):1336. doi: 10.3390/vaccines9111336 (PMC8620474; doi:10.3390/vaccines9111336)
Supplement: Supplementary file 1 [file vaccines-09-01336-s001.zip › vaccines-1448671-supplementary.pdf]

Supplementary Material Table S1. Translated version of the study questionnaire.

Questions	Answers
**Year of birth of the respondent**	• Numeric
**Sex of the respondent**	• Male<br>• Female
**Place of residence**	• Please declare
**M1. Do you currently work for profit (full-time, in your own company or farm, or do you undertake commissioned work)?**	• Yes, part-time<br>• Yes, full time<br>• Yes, casual<br>• Not
**M4. What is your education? Please provide the highest level of education achieved by you.**	• elementary / junior high school<br>• -basic vocational<br>• secondary<br>• higher
**M5. How many people, including yourself, does your household consist of?**	• Numeric
**M6. How many adults, i.e. those aged 18 or over, make up your household? Please include yourself as well.**	• Numeric
**M7. How do you assess the current material conditions of your household? Are they:**	• bad<br>• rather bad<br>• neither good nor bad<br>• rather good<br>• good
**M14. Regardless of your participation in religious practices, do you consider yourself as person:**	• deeply believer<br>• rather unbeliever<br>• completely unbeliever<br>• refusal to answer
**M17. Do you have an e-mail that you check regularly - i.e. at least 3-4 times a week?**	• Yes<br>• No
**Have you been vaccinated against influenza this fall / winter season?**	• Yes<br>• No<br>• Refusal to answer
**Are you going to have influenza vaccine this fall / winter season?**	• Yes<br>• No<br>• Refusal to answer

**24. Are you personally afraid of coronavirus infection?**	• Yes, I am very afraid<br>• Yes, I'm a little scared<br>• No, I'm not afraid<br>• No, I'm not afraid at all<br>• Hard to say
**36. In your opinion, do you think the coronavirus epidemic for the health of Poles:**	• is a real threat<br>• is an exaggerated threat<br>• it is a fictional threat at all<br>• hard to say
**26. Why would you not want to be vaccinated against COVID-19?**	• I am concerned about the side effects of the COVID-19 vaccine<br>• I am concerned that the COVID-19 vaccine will not be effective<br>• I avoid vaccinations at all<br>• I have already had COVID-19<br>• I believe that COVID-19 is not a serious disease<br>• Other reasons
**37. Pharmaceutical lobbies, politicians and the media around the world are deliberately exaggerating the dangers of the coronavirus**	• I definitely agree<br>• I tend to agree<br>• I rather disagree<br>• I strongly disagree<br>• Hard to say
**38. The coronavirus pandemic was artificially triggered to reduce the population of humanity on Earth**	• I definitely agree<br>• I tend to agree<br>• I rather disagree<br>• I strongly disagree<br>• Hard to say
**42. If a COVID-19 vaccine was available, would you get vaccinated against the disease?**	• I definitely agree<br>• I tend to agree<br>• I rather disagree<br>• I strongly disagree<br>• Hard to say
